# Retraction: Up-Regulation of Intestinal Epithelial Cell Derived IL-7 Expression by Keratinocyte Growth Factor through STAT1/IRF-1, IRF-2 Pathway

**DOI:** 10.1371/journal.pone.0305938

**Published:** 2024-06-18

**Authors:** The PLOS ONE Editors

Following the publication of this article [1], concerns were raised regarding results presented in Figures 6 and 9. Specifically,

- The Figure 9A H1 panel and the Figure 9A Tubulin panel appear similar. In addition, lanes 1–4 of these panels appear similar to the Figure 6B Tubulin panel despite representing different experimental conditions.
- The Figure 9B H1 panel appears similar to the Figure 9B Tubulin panel when flipped horizontally.
- Figure 9C appears to be missing a control group (Plasmid control + KGF) needed to support the figure’s results.

The journal discussed the panel similarity concerns detected in Figure 9 with the authors previously. The authors stated that the images were mislabelled which led to the duplication of the panels during figure preparation; they provided replacement H1 panels for Figures 9A and B. Uncropped underlying data for these figures were not provided.

The authors did not respond to recent editorial communications, which included questions about the other issues listed above.

In the absence of the original uncropped underlying data for Figures 6 and 9, the concerns cannot be resolved.

In light of the above concerns that question the reliability of the results presented in Figures 6 and 9, the *PLOS ONE* Editors retract this article.

All authors either did not respond directly or could not be reached.
